# Plant genome information facilitates plant functional genomics

**DOI:** 10.1007/s00425-024-04397-z

**Published:** 2024-04-09

**Authors:** Judith Jazmin Bernal-Gallardo, Stefan de Folter

**Affiliations:** Unidad de Genómica Avanzada (UGA-Langebio), Centro de Investigación y de Estudios Avanzados del Instituto Politécnico Nacional (Cinvestav), Irapuato, Mexico

**Keywords:** Plant genomes, Plant development, Genomics, Sequencing, Genes

## Abstract

**Main conclusion:**

In this review, we give an overview of plant sequencing efforts and how this impacts plant functional genomics research.

**Abstract:**

Plant genome sequence information greatly facilitates the studies of plant biology, functional genomics, evolution of genomes and genes, domestication processes, phylogenetic relationships, among many others. More than two decades of sequencing efforts have boosted the number of available sequenced plant genomes. The first plant genome, of Arabidopsis, was published in the year 2000 and currently, 4604 plant genomes from 1482 plant species have been published. Various large sequence initiatives are running, which are planning to produce tens of thousands of sequenced plant genomes in the near future. In this review, we give an overview on the status of sequenced plant genomes and on the use of genome information in different research areas.

**Supplementary Information:**

The online version contains supplementary material available at 10.1007/s00425-024-04397-z.

## Introduction

**Fig. 1:**
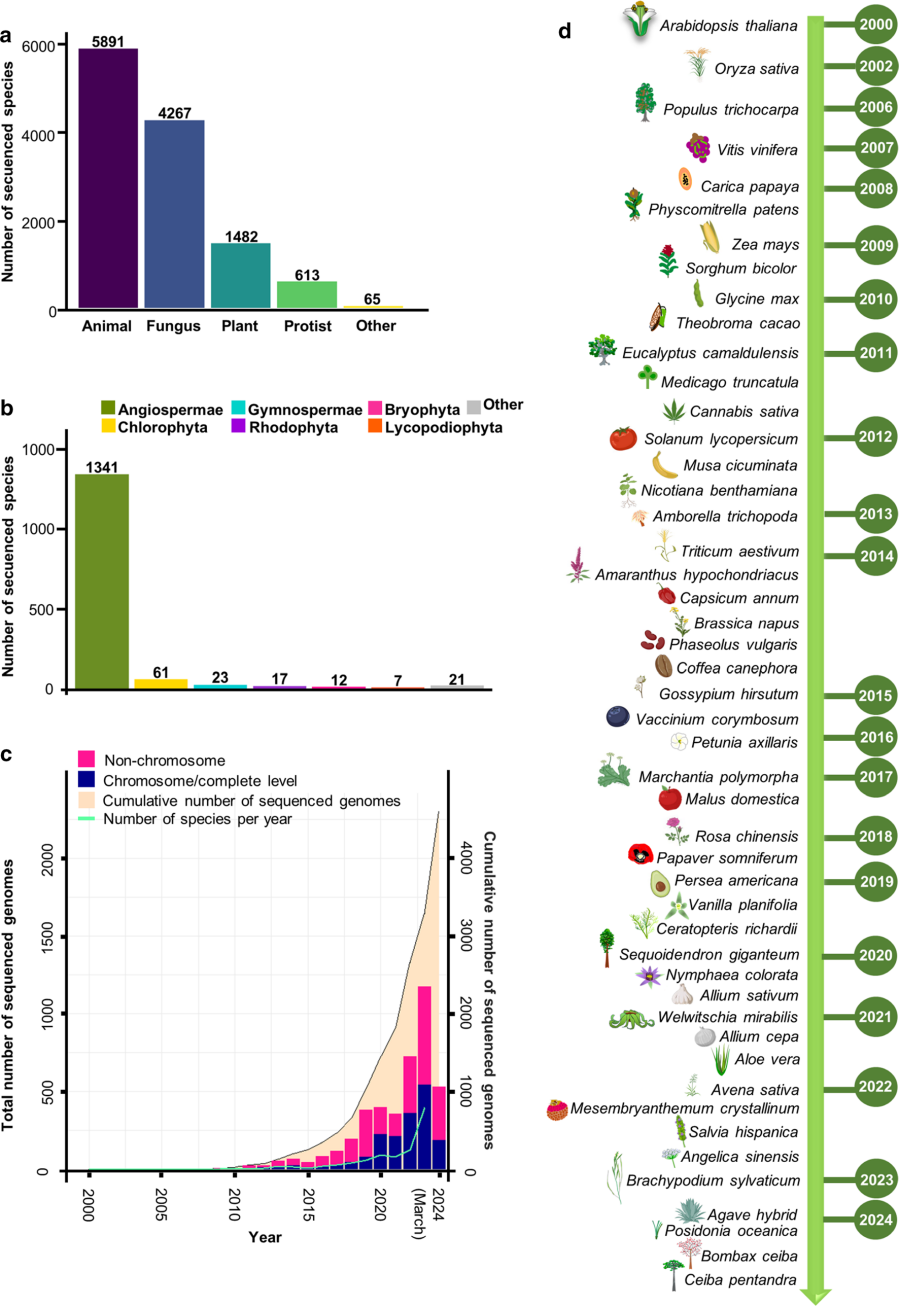
Sequenced genomes of plant species.** a** The plant kingdom stands as the third-most sequenced domain of life, as evidenced by the cumulative number of sequenced species. **b** Boxplot of sequenced species across the main clades of the Plant Kingdom. **c** Graphical representation of the progression in plant genome sequencing since 2000. The bars illustrate the distribution of plant genomes at both chromosomal and non-chromosomal levels. The green line tracks the annual sequencing rate of species, while the salmon shadowed area represents the cumulative count of sequences through March 2024. For the latter two, use values on the right y-axis. **d** Chronology of sequenced key plants of agriculturally and scientifically important plant species. In **a**, the data for animals, fungi, protists, and other domains of life were acquired from the NCBI database (https://www.ncbi.nlm.nih.gov/). Sequenced plant species counts were obtained from https://www.plabipd.de/, with the information updated on 19 February 2024. In **b**, species count data and genome sequencing details at both chromosomal and non-chromosomal levels were obtained from the NCBI database. Species counts were verified and updated using information from https://www.plabipd.de/. In (**c**), the chronology was constructed using data obtained from the NCBI database and some of the images were generated using BioRender.com

The blueprint of living organisms sits in its DNA. It contains the instructions for an organism to grow and develop. In the last two decades, genome sequencing has greatly advanced. Currently, the NCBI database (https://www.ncbi.nlm.nih.gov/) holds information on 30,530 eukaryotic genomes (representing 12,205 species), of which 5119 are complete or at chromosome level (accessed on 5 March 2024; Fig. 1). From these sequencing efforts, it became clear that the complexity of an organism is not necessary in the number of its genes. For instance, the number of genes of human (International Human Genome Sequencing Consortium 2001; Venter et al. 2001) or a roundworm (C. elegans Sequencing Consortium 1998) are not that far apart. A big part of the complexity is in how gene expression is regulated, and finally in how many proteins this can result. Genome information drives the discovery of biological insights on how organisms are functioning and their evolutionary history, and as well for biotechnological innovations. In the field of agriculture, genome information helps modern breeding, facilitates climate adaptation and food security, among others. Though it does not stop here, genome sequence efforts continue around the world. To highlight one large effort, the Earth BioGenome Project, which aims to sequence every living eukaryotic organism with a name on our planet, which is around 2 million species (Lewin et al. 2018; Ebenezer et al. 2022). A genomic tree of life is intended to aid in our understanding of how species change, adapt, and rely on one another across an ecosystem. Through these discoveries, long-standing problems in phylogenetics, evolution, ecology, conservation, agriculture, the bioindustry, and medicine will be resolved (Blaxter et al. 2022).

In this review, we give an overview of the status of (nuclear) plant genome sequencing efforts and how this has helped for studies on plant functional genomics.

## The status of sequenced plant genomes

Information on plant genome sequences enormously facilitates studies on plant biology, genetics, development, evolution, molecular biology, among many others. The first sequenced plant genome, *Arabidopsis thaliana*, was published in the year 2000 (Arabidopsis Genome Initiative 2000). This model plant is widely used worldwide and with the genome sequence, it opened the plant field into the genomics era. For a historical overview of Arabidopsis, we refer to other reviews (Meyerowitz 2001; Provart et al. 2016, 2021; Somssich 2019). Arabidopsis has a genome size of around 135 Mb, and based on the latest Araport11 re-annotation, has 27,655 protein-coding loci with 48,359 transcripts (Cheng et al. 2017). Various dedicated websites house data for the community such as The Arabidopsis Information Resource (TAIR; Rhee et al. 2003), Araport (Cheng et al. 2017; Pasha et al. 2020), ThaleMine (Krishnakumar et al. 2017; Pasha et al. 2020), and Bio-Analytic Resource (BAR; Toufighi et al. 2005).

**Fig. 2:**
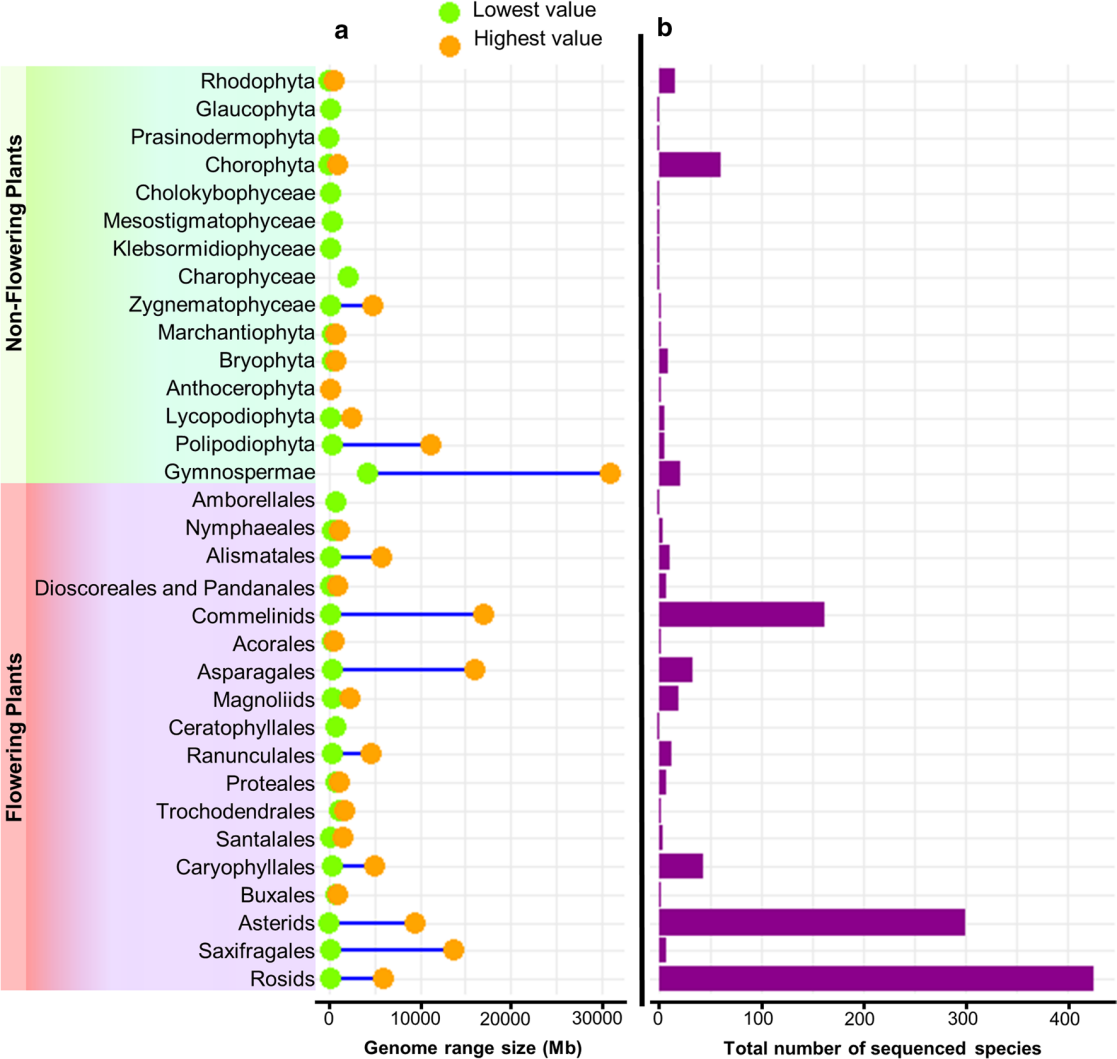
Genome size and species count across plant clades.** a** Range of genome size within each clade of plant classification, with data points denoting the minimum and maximum genome sizes for each clade. **b** Bars illustrating the distribution of the number of species within each clade of plant classification. The plant classification used is based on the taxonomy provided by https://www.plabipd.de/, which was updated on 19 February 2024

Nowadays, plant genome sequencing is a very active field (Michael and Jackson 2013; Chen et al. 2018; Kersey 2019; Marks et al. 2021; Kress et al. 2022; Sun et al. 2022). Since the publication of the Arabidopsis genome in December 2000 (Arabidopsis Genome Initiative 2000) 4604 nuclear plant genomes have been sequenced, corresponding to 1482 plant species, most of them being from angiosperms (90%) (Figs. 1 and 2). This genome data are based on information from the NCBI database (accessed on 5 March 2024; https://www.ncbi.nlm.nih.gov/genome/browse#!/overview/plants), and from the website *Published Plant Genomes* that visualizes sequenced plant genomes over time (https://www.plabipd.de/; R. Schwacke, personal communication, 19 February 2024). The second plant species to have a genome sequenced was rice, with two subspecies of rice (*Oryza Sativa* subsp. *japonica* and subsp. *indica*; Goff et al. 2002; Yu et al. 2002); in 2006 the first genome of a tree, from poplar (*Populus trichocarpa*; Tuskan et al. 2006); and in 2007 the genome of grape, the first genome of a fruit producing species (*Vitis vinifera*; Velasco et al. 2007). In the second decade of sequencing, the number of genome reports per year went up exponentially (Fig. 1).

**Fig. 3:**
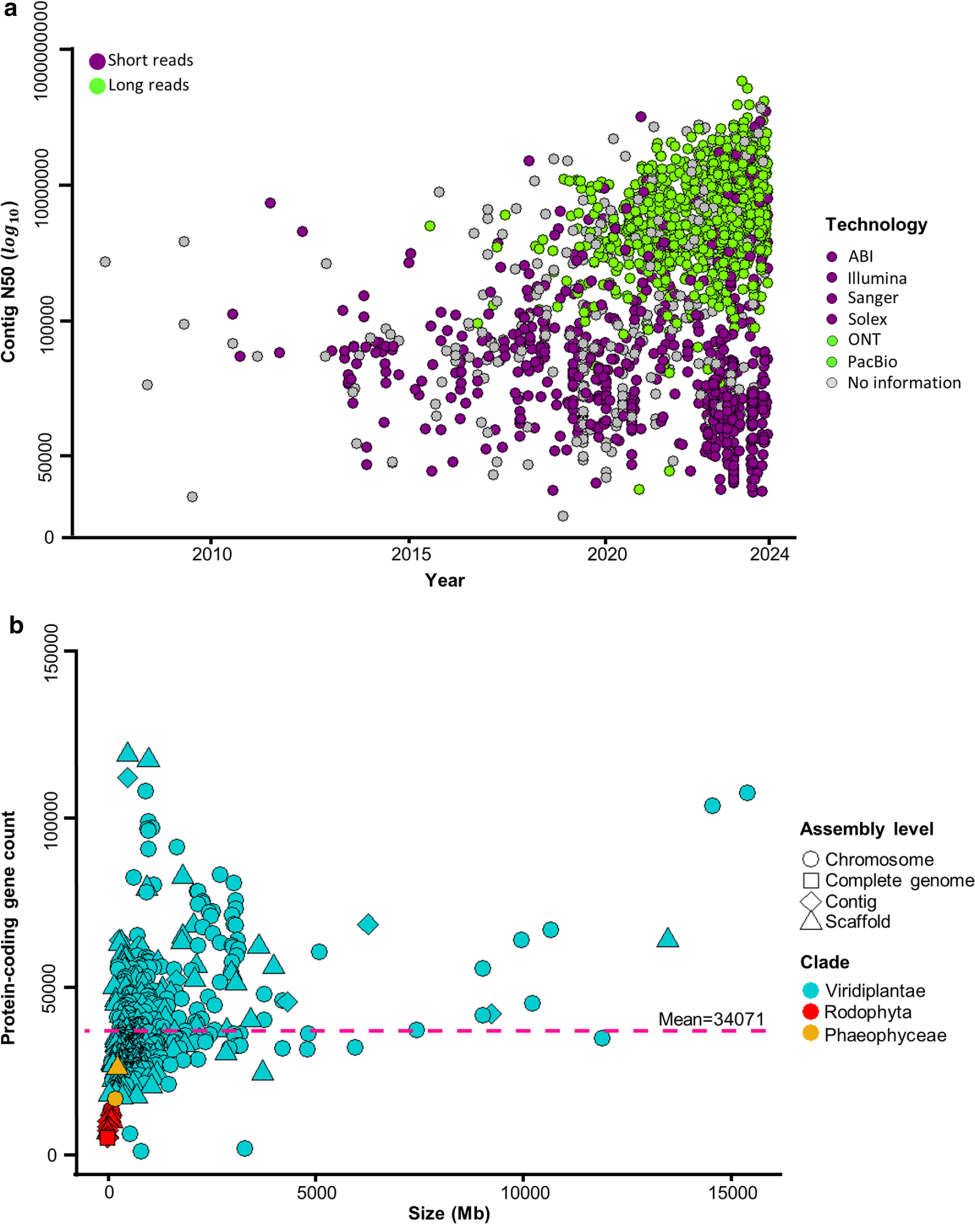
Comparative analysis of genome size and protein-coding genes in annotated plant genomes, and assembly statistics of contig N50 over time for sequenced plant species. **a** Distribution of assembly statistics: Contig N50 over time for the 1482 sequenced plant species; data obtained from the NCBI Database (https://www.ncbi.nlm.nih.gov/). The green points represent assemblies based on long-read sequencing methods, while the purple points represent assemblies based on short-read sequencing methods. **b** The graph illustrates the distribution of the genome size and the number of protein-coding genes (the pink dashed line indicates the mean number of genes per genome: 34,071) in the 685 available annotated plant genomes, utilizing taxonomic classifications from the NCBI database (https://www.ncbi.nlm.nih.gov/). Points are colored by assembly level, and the figure represents a clade of the Plant Kingdom

Just in the last five year, numbers of sequenced nuclear plant genomes increased impressively from around 576 (reflecting 383 species) (Kersey 2019), 798 (reflecting 798 species) (Marks et al. 2021), 1031 (reflecting 788 species) (Sun et al. 2022), 1139 (reflecting 812 species) (Kress et al. 2022), to 4604 genome sequences (reflecting 1482 species) that have been reported (5 March 2024; Table S1). This has to do with improvements of sequence technologies and lower costs (Shendure et al. 2017; Michael and VanBuren 2020; Henry 2022). One of the descriptions of the quality of genome assemblies is the value of the Contig N50, which indicates the length of the shortest contig in the set of contigs containing at least 50% of the assembly length. This value greatly improved over the years (Fig. 3a), which is low (< 1 kb or < 10 kb) when a short-read sequencing approach was used (e.g., Illumina), and nowadays, with the use of long-read sequencing approaches such as from Pacific Biosciences (PacBio) and Oxford Nanopore Technologies (ONT), the Contig N50 is hundreds of kb to several Mb, resulting in much higher quality genome assemblies (Michael and Jackson 2013; Belser et al. 2018; Kersey 2019; Michael and VanBuren 2020; Marks et al. 2021; Sharma et al 2021; Sun et al. 2022).

The estimated number of extant green plant species is around 450,000–500,000 (Corlett 2016; Lughadha et al. 2016). The number of green plant species with sequenced genomes (1482) represents around 0.26–0.29% of plant species, so only a fraction of them has been sequenced so far. Despite an uneven distribution, the reported genomes span around 500 million years of evolution and comprise the major clades of green plants (Viridiplantae) (Fig. 2). Nuclear plant genome size varies greatly among the sequenced species, from 9 Mb to 31 Gb (Fig. 2). In contrast to more than 3000-fold difference in genome size, the number of protein-coding genes per genome varies much less, only in the range of a few-fold difference (Fig. 3b). Based on the 685 available annotated plant genomes depicted in Fig. 3b, the mean number of protein-coding genes is 34,071 (Table S1). Large genome sizes are attributed in part to polyploidy events common in plants, but mainly to the activity of transposable elements (Michael and Jackson 2013; Michael 2014; Kersey 2019; Kress et al. 2022; Marks et al. 2021).

Furthermore, we can see that the model species and many agriculturally and economically important plant species have been sequenced (Figs. 1 and 2). Without doubt, the number of sequenced genomes and phylogenetic distributions of them will soon increase and expand, because of many current genome initiatives. Projects affiliated to the Earth BioGenome Project (Lewin et al. 2018, 2022), is the Darwin Tree of Life Project that aims to sequence all 70,000 species in Britain and Ireland (Darwin Tree of Life Project Consortium 2022). Another example is the 10KP (10,000 Plants) Initiative, which aims to sequence genomes of 10,000 species representing every major clade of embryophytes (land plants), green algae (chlorophytes and streptophytes), and protists (photosynthetic and heterotrophic) (Cheng et al. 2018). Other initiatives are the African BioGenome Project (AfricaBP) aiming to sequence genomes of 105,000 endemic species, including plants (Ebenezer et al. 2022), the African Orphan Crops Consortium (AOCC) aiming to sequence 101 African orphan crops/trees (Hendre et al. 2019), and the Genomics for Australian Plants (GAP) consortium aiming to sequence representative Australian plant genomes across the plant tree of life (Genomics for Australian Plants Initiative 2018; McLay et al. 2022).

Mostly, when sequencing a genome, the genome of one individual species is sequenced, which will be used as the reference genome. However, this is unlikely to be the complete picture. Genetic differences among individual species may exist. To overcome this, the term pan-genome was coined. The first report was based on the sequencing of eight bacterial strains and the observation that not every gene was present in each strain (Tettelin et al. 2005). It refers to the ´whole´ genome within a species (Golicz et al. 2020; Bayer et al. 2020). A pan-genome can be made by sequencing different individuals, accessions, cultivars, or populations, and then by ´joining´ the information, the whole genetic diversity will be captured, in principle (Lei et al. 2021; Li et al. 2022). In plants, the first pan-genome was made for wild soybean (*Glycine soja*), by sequencing and de novo assembly of seven phylogenetically and geographically representative accessions (Li et al. 2014). To date, around 30 plant pan-genomes, mostly of crops, have been published (Li et al. 2022). To create pan-genomes, long read sequencing is used. Normally, for re-sequencing efforts, short read sequencing is used, which allows the detection of single nucleotide polymorphisms (SNPs), but structural variants (SVs) are more difficult to identify (Golicz et al. 2020).

For comparative plant genomics, we refer readers to the useful website Phytozome (Goodstein et al. 2012).

## How plant genomes facilitate plant functional genomics

### Gene function discovery using mutant collections

With the availability of genome sequences, the identification of gene functions via mutant screens became much easier. To go from a phenotype to the probable casual mutation induced by ethyl methanesulfonate (EMS) mutagenesis using classical forward genetic screens involved long and laborious mapping strategies. Nowadays, mapping can be performed by sequencing the genomes of a population of backcrossed homozygous plants with the phenotype of interest, which allows the rapid identification of the casual mutation (Hartwig et al. 2012; Garcia et al. 2016).

In reverse genetic screens, starting with a gene of interest and determining the phenotype/function (Alonso and Ecker 2006), for 20 years the Arabidopsis community has used insertional T-DNA mutant collections where sequence information is available for most of the random T-DNA insertions in the genome, arguably, the most widely used is the SALK T-DNA collection (Alonso et al. 2003). Various other valuable sequenced collections of T-DNA, transposon insertion, or variations, are available for Arabidopsis (Samson et al. 2002; Sessions et al. 2002; Rosso et al. 2003; Woody et al. 2007), and for other model species such as rice (Wang et al. 2013; Wei et al. 2013), maize (Lu et al. 2018), and petunia (Vandenbussche et al. 2008, 2016).

There are various other techniques available for gene function discovery where genome information is very useful. An example of a reverse genetics approach to find mutations is TILLING (Targeting Induced Local Lesions IN Genomes), which is a chemical random mutagenesis approach, followed by high-throughput screening of point mutations in targeted genomic regions. The screening part can be combined with high-throughput sequencing (Mccallum et al. 2000; Henikoff et al. 2004; Tadele 2016). Another frequently used approach is activation tagging to identify gain-of-function mutants. For this, a mutant population is made by random genome insertions of T-DNAs or transposons carrying an activation sequence, leading to the activation of nearby genes. Recovering the flanking sequence followed by the identification of the genome region leads to the discovery of the gene in question (Weigel et al. 2000; Marsch-Martinez et al. 2002; Tani et al. 2004).

Other reverse genetics approaches for gene function discovery, involve making dedicated constructs targeting genes of interests, which can be used to target one or more genes. RNA interference (RNAi) (Saurabh et al. 2014; Muhammad et al. 2019) or the fusion of a transcriptional repression domain (EAR domain) (Hiratsu et al. 2003; Mitsuda et al. 2011) can be used to obtain loss-of-function mutants. Another approach is the use of artificial miRNAs (amiRNAs) to silence genes. An amiRNA can be designed to silence one gene or a family of redundant genes (Schwab et al. 2006; Ossowski et al. 2008). A last example of an approach, still relatively new but already very actively used, is using a CRISPR-Cas system (Wada et al. 2020; Zhu et al. 2020; Gaillochet et al. 2021). The used guide RNAs (gRNAs) are typically directed towards coding regions, but can also be directed towards promoters or non-coding regions. Furthermore, multiple gRNAs can be cloned in the same vector to target different genes (Najera et al. 2019) or promoters (Rodríguez-Leal et al. 2017). Having the genome information, genome-wide screens can be made using pooled CRISPR libraries (Huang et al. 2022; Liu et al. 2023; Pan et al. 2023), and various reports have already been published such as in rice (Lu et al. 2017; Meng et al. 2017), tomato (Jacobs et al. 2017), soybean (Bai et al. 2020), maize (Liu et al. 2020), and canola (He et al. 2023).

The use of CRISPR systems, for ´traditional´ genome editing or for gene activation/repression, may fill the gap of functional genomics in plant species, beyond the model species currently used (Huang et al. 2022; Liu et al. 2023; Pan et al. 2023). With the use of pooled CRISPR libraries, massive plant transformation could be applied in different species. Sharing of whole genome gRNA library data, pooled libraries, and even complete transformed CRISPR mutant populations in the form of seeds could make a usage boost to functional studies. As mentioned above, 4,604 nuclear plant genomes have been sequenced, corresponding to 1482 plant species (Fig. 1), most functional genomics research is performed in a rough estimate of only 1–2% of plant species with genome information so far. The future holds interesting opportunities for the use of genome information.

### OMICS technologies

In addition to genomics, there are now many other *omics* technologies available. All these technologies benefit greatly from genome information. Many efforts exist generating plant transcriptomes from model species but also non-model species, even from species with no genome information yet. For the latter, mapping of the sequence reads is done against the genome of the evolutionary closest species or reads can be mapped (and gene expression quantified) against a de novo assembled transcriptome from the target organism. In general, transcriptome information also helps to improve genome annotations. Many databases exist to explore transcriptome data such as BAR (Winter et al. 2007), Genevestigator (Zimmermann et al. 2004), and Plant Public RNA-seq Database (Yu et al. 2022). Other databases contain data from large initiatives like the 1KP (1000 Plants), where transcriptomes of 1124 species were sequenced to infer the phylogenomic relationships (Matasci et al. 2014; Leebens-Mack et al. 2019). Another initiative is the JGI Plant Gene Atlas, which contains almost 2100 RNA-Seq data sets collected from 18 plant species, with the aim to improve functional gene descriptions across the plant kingdom (Sreedasyam et al. 2023). Recently, a great number of specialized single cell and single nuclei transcriptome data sets are emerging (reviewed in: Seyfferth et al. 2021; Cervantes-Perez et al. 2022; Denyer and Timmermans 2022; Nolan and Shahan 2023; Zheng et al. 2023) and databases holding single cell transcriptome data (e.g., Ma et al. 2020; Wendrich et al. 2020; Chen et al. 2021a; He et al. 2023).

Plant proteomics is also a large field and benefits from genome information, including transcriptome information, first to be able to predict all proteins and isoforms (Chen et al. 2021a, b; Mergner and Kuster 2022). Many proteomic studies, from small studies to very large studies, and even pan-plant proteomes have been reported in the literature (e.g., McWhite et al. 2020; Mergner et al. 2020; van Wijk et al. 2021, 2024).

An *omics* area that has a growing significance that can improve draft plant genomes, correct gene annotation, discover new translation initial sites, ORFs, and alternative splicing, and verify novel genes of the peptide/protein level is called proteogenomics (Nesvizhskii 2014; Song et al. 2023). The usefulness of proteogenomics has been illustrated for instance for the model organism Arabidopsis (e.g., Castellana et al. 2008; Zhu et al. 2017; Willems et al. 2017, 2022). Recent examples of proteogenomics in other species are for sweet cherry and pear (Xanthopoulou et al. 2021; Wang et al. 2023).

Another big *omics* technology is metabolomics. Metabolomics is a good tool for functional genomics (Schauer and Fernie 2006). It is a powerful technique to analyze the metabolite content in plants and is less restricted to genome information or model species. Though limitations for metabolomics in some (non-model) plants are the lack of high-quality metabolite databases, such that some molecules cannot easily be unambiguously identified. On the other hand, combining different types of *omics* data can lead to the discovery of gene functions and help in future plant improvements (Kumar et al. 2017; Patel et al. 2021; Shen et al. 2023).

### Evolution and domestication

Genome information facilitates the study of phylogenetic relationships among species. Furthermore, the importance of genes or gene families in the evolution of land plants can be studied (Yu et al. 2018; Leebens-Mack et al. 2019; Soltis and Soltis 2021; Guo et al. 2023). Another example facilitated by genome information is the study of domestication. Hundreds of plant species have been domesticated by humans by selecting for beneficial traits (Gepts 2004; Meyer and Purugganan 2013). Through candidate gene studies, quantitative trait locus (QTL) mapping and cloning, genome-wide association studies (GWASs), and whole-genome resequencing studies, a significant number of domestication or domestication-related genes have been discovered and isolated (Meyer and Purugganan 2013; Kantar et al. 2017). More recently, reports on pan-genomes also facilitate the study of evolution and domestication, and the identification of key genes associated with important agronomic traits (Li et al. 2022).

Interestingly, de novo domestication by genome editing has been used (Bartlett et al. 2023). For instance, using CRISPR-Cas9, this has been done in the wild tomato species (Li et al. 2018; Zsögön et al. 2018), in the Solanaceae species ´groundcherry´ (Lemmon et al. 2018), and in wild rice (Yu et al. 2021). Knowledge on domesticated genes was used to edit several of these genes at once, resulting directly in a ´crop´ with desirable agricultural traits.

## Conclusion and perspective

In recent years, the number of sequenced plant genomes has increased at an incredible speed. It is clear that this will only continue, and in the near future we will have tens of thousands of sequenced plant genomes. This wealth of information will accelerate studies on plant biology, functional genomics, evolution of genomes and genes, domestication processes, phylogenetic relationships, among many others. In parallel, new and improved bioinformatics analysis methods will have to be developed.

The field of single cell genomics will also expand and will also come with technical challenges such as capturing more cells, capturing low-abundance cells, cell-type annotation, new sequencing and analysis methods (Efroni and Birnbaum 2016; Conde and Kirst 2022; Cuperus 2022). Moreover, this will not only apply to transcriptomics, but in all *omics* fields we are going to see a rapid expansion, from single cell *omics*, single cell multi-*omics*, spatial genomics and other *omics*, new *omics* analysis methods, and to inference of gene regulatory networks using single cell *omics* data, among others (Thibivilliers and Libault 2021; Clark et al. 2022; Yu et al. 2023; Baysoy et al. 2023).

The genome evolution and phylogenomic research field will have an ever-growing amount of data available for analyses. Furthermore, there is a great potential for the use of functional genomics data for genome-editing of crops and for the de novo domestication for future crops using this same technology (Fernie and Yan 2019; Zhou et al. 2020; Zaidi et al. 2020; Gao 2021; Kumar et al. 2022; Yu and Li 2022; Bartlett et al. 2023). Importantly, when it comes to crop yield, knowledge is required how to properly evaluate this (Khaipho-Burch et al. 2023).

Lastly, Artificial Intelligence (AI) is certainly going to play a role in the plant science fields discussed here. Predictive models or analysis methods are developed based on machine learning (ML) and deep learning (DL) (Wang et al. 2020; van Dijk et al. 2021; Xu et al. 2021; Holzinger et al. 2023). Besides ChatGPT as a tool to ask or write texts, among other tasks (OpenAI; https://chat.openai.com/chat), probably one of the best-known tools now in life sciences, is AlphaFold and its successor Alphafold2, a model that can predict almost all protein tertiary structures (Senior et al. 2020; Jumper et al. 2021). Other examples are the use of AI in image analysis and image-based phenotyping, having autonomous robots and/or drones for plant phenotyping, pest management, fertilizer management, or harvesting (Harfouche et al. 2023; Holzinger et al. 2023; Murphy et al. 2024). Furthermore, AI can be applied in bioinformatic analysis, to improve genome annotations, predict with high accuracy specific motifs in regulatory regions, gene function prediction, or predict the import nucleotide region or gene(s) in EMS screens or QTL analysis, etc. These are just a few examples of the many possibilities of the use of AI now and in the near future.

In conclusion, plant genomics will undoubtedly remain a cornerstone, actively contributing to the ongoing advancement of plant science and its practical applications.

### Supplementary Information

Below is the link to the electronic supplementary material.

Supplementary file1 (XLSX 723 KB)

## Data Availability

The datasets generated during and/or analyzed during the current study are available from the corresponding author upon reasonable request.

## Note added in proof

The article by Xie et al. (2024) was published where they present the N3 database on plant genome sequence efforts: http://ibi.zju.edu.cn/N3database/index.php.

## References

[CR1] Alonso JM, Ecker JR (2006). Moving forward in reverse: Genetic technologies to enable genome-wide phenomic screens in Arabidopsis. Nat Rev Genet.

[CR2] Alonso JM, Stepanova AN, Leisse TJ, Kim CJ, Chen H, Shinn P (2003). Genome-Wide Insertional Mutagenesis of *Arabidopsis thaliana*. Science.

[CR3] Arabidopsis Genome Initiative (2000). Analysis of the genome sequence of the flowering plant *Arabidopsis thaliana*. Nature.

[CR4] Bai M, Yuan J, Kuang H, Gong P, Li S, Zhang Z (2020). Generation of a multiplex mutagenesis population via pooled CRISPR-Cas9 in soya bean. Plant Biotechnol J.

[CR5] Bartlett ME, Moyers BT, Man J (2023). The power and perils of De Novo domestication using genome editing. Annu Rev Plant Biol.

[CR6] Bayer PE, Golicz AA, Scheben A (2020). Plant pan-genomes are the new reference. Nat Plants.

[CR7] Baysoy A, Bai Z, Satija R, Fan R (2023). The technological landscape and applications of single-cell multi-omics. Nat Rev Mol Cell Biol.

[CR151] Belser C, Istace B, Denis E (2018). Chromosome-scale assemblies of plant genomes using nanopore long reads and optical maps. Nature Plants.

[CR8] Blaxter M, Archibald JM, Childers AK (2022). Why sequence all eukaryotes?. Proc Natl Acad Sci U S A.

[CR9] C. elegans Sequencing Consortium (1998). Genome Sequence of the Nematode *C. elegans*: a platform for investigating biology. Science.

[CR10] Castellana NE, Payne SH, Shen Z (2008). Discovery and revision of Arabidopsis genes by proteogenomics. Proc Natl Acad Sci U S A.

[CR11] Cervantes-Pérez SA, Thibivillliers S, Tennant S, Libault M (2022). Review: Challenges and perspectives in applying single nuclei RNA-seq technology in plant biology. Plant Sci.

[CR12] Chen F, Dong W, Zhang J (2018). The sequenced angiosperm genomes and genome databases. Front Plant Sci.

[CR13] Chen H, Yin X, Guo L (2021). Plant scRNAdb: A database for plant single-cell RNA analysis. Mol Plant.

[CR14] Chen Y, Wang Y, Yang J (2021). Exploring the diversity of plant proteome. J Integr Plant Biol.

[CR15] Cheng CY, Krishnakumar V, Chan AP (2017). Araport11: a complete reannotation of the *Arabidopsis thaliana* reference genome. Plant J.

[CR16] Cheng S, Melkonian M, Smith SA (2018). 10KP: a phylodiverse genome sequencing plan. Gigascience.

[CR17] Clark NM, Elmore JM, Walley JW (2022). To the proteome and beyond: advances in single-cell omics profiling for plant systems. Plant Physiol.

[CR18] Conde D, Kirst M (2022). Decoding exceptional plant traits by comparative single-cell genomics. Trends Plant Sci.

[CR19] Corlett RT (2016). Plant diversity in a changing world: Status, trends, and conservation needs. Plant Divers.

[CR20] Cuperus JT (2022). Single-cell genomics in plants: current state, future directions, and hurdles to overcome. Plant Physiol.

[CR21] Darwin Tree of Life Project Consortium (2022). Sequence locally, think globally: The Darwin Tree of Life Project. PNAS.

[CR22] Denyer T, Timmermans MCP (2022). Crafting a blueprint for single-cell RNA sequencing. Trends Plant Sci.

[CR23] Ebenezer TE, Muigai AWT, Nouala S (2022). Africa: sequence 100,000 species to safeguard biodiversity Setting the agenda in research. Nature.

[CR24] Efroni I, Birnbaum KD (2016). The potential of single-cell profiling in plants. Genome Biol.

[CR25] Fernie AR, Yan J (2019). De novo domestication: an alternative route toward new crops for the future. Mol Plant.

[CR26] Gaillochet C, Develtere W, Jacobs TB (2021). CRISPR screens in plants: approaches, guidelines, and future prospects. Plant Cell.

[CR27] Gao C (2021). Genome engineering for crop improvement and future agriculture. Cell.

[CR28] Garcia V, Bres C, Just D (2016). Rapid identification of causal mutations in tomato EMS populations via mapping-by-sequencing. Nat Protoc.

[CR29] Genomics for Australian Plants Initiative (2018) 10.25953/3108-3v82

[CR30] Gepts P, Janick J (2004). Crop domestication as a long-term selection experiment. Plant breeding reviews.

[CR31] Goff SA, Ricke D, Lan T-H (2002). A draft sequence of the rice genome (*Oryza sativa* L. ssp. japonica). Science.

[CR32] Golicz AA, Bayer PE, Bhalla PL (2020). Pangenomics comes of age: from bacteria to plant and animal applications. Trends Genet.

[CR33] Goodstein DM, Shu S, Howson R, et al (2012) Phytozome: A comparative platform for green plant genomics. Nucleic Acids Res 40. 10.1093/nar/gkr944 10.1093/nar/gkr944 PMC3245001 22110026

[CR34] Guo C, Luo Y, Gao LM, Yi T (2023). Phylogenomics and the flowering plant tree of life. J Integr Plant Biol.

[CR35] Harfouche AL, Nakhle F, Harfouche AH (2023). A primer on artificial intelligence in plant digital phenomics: embarking on the data to insights journey. Trends Plant Sci.

[CR36] Hartwig B, James GV, Konrad K (2012). Fast isogenic mapping-by-sequencing of ethyl methanesulfonate-induced mutant bulks. Plant Physiol.

[CR37] He Z, Luo Y, Zhou X, Zhu T, Lan Y, Chen D (2023). scPlantDB: a comprehensive database for exploring cell types and markers of plant cell atlases. Nucleic Acids Res.

[CR38] Hendre PS, Muthemba S, Kariba R (2019). African Orphan Crops Consortium (AOCC): status of developing genomic resources for African orphan crops. Planta.

[CR39] Henikoff S, Till BJ, Comai L (2004). TILLING. Traditional mutagenesis meets functional genomics. Plant Physiol.

[CR40] Henry RJ (2022). Progress in plant genome sequencing. Appl Biosci.

[CR41] Hiratsu K, Matsui K, Koyama T, Ohme-Takagi M (2003). Dominant repression of target genes by chimeric repressors that include the EAR motif, a repression domain, in Arabidopsis. Plant J.

[CR42] Holzinger A, Keiblinger K, Holub P (2023). AI for life: trends in artificial intelligence for biotechnology. N Biotechnol.

[CR43] Huang Y, Shang M, Liu T, Wang K (2022). High-throughput methods for genome editing: the more the better. Plant Physiol.

[CR44] International Human Genome Sequencing Consortium (2001). Initial sequencing and analysis of the human genome. Nature.

[CR45] Jacobs TB, Zhang N, Patel D, Martin GB (2017). Generation of a collection of mutant tomato lines using pooled CRISPR libraries. Plant Physiol.

[CR46] Jumper J, Evans R, Pritzel A (2021). Highly accurate protein structure prediction with AlphaFold. Nature.

[CR47] Kantar MB, Nashoba AR, Anderson JE (2017). The genetics and genomics of plant domestication. Bioscience.

[CR48] Kersey PJ (2019). Plant genome sequences: past, present, future. Curr Opin Plant Biol.

[CR49] Khaipho-Burch M, Cooper M, Crosssa J, de Leon N, Holland James LR (2023). Scale up trials to validate modified crops’ benefits. Nature.

[CR50] Kress WJ, Soltis DE, Kersey PJ (2022). Green plant genomes: what we know in an era of rapidly expanding opportunities. PNAS.

[CR51] Krishnakumar V, Contrino S, Cheng CY, Belyaeva I, Ferlanti ES, Miller JR (2017). Thalemine: a warehouse for Arabidopsis data integration and discovery. Plant Cell Physiol.

[CR52] Kumar R, Bohra A, Pandey AK (2017). Metabolomics for plant improvement: Status and prospects. Front Plant Sci.

[CR53] Kumar K, Mandal SN, Pradhan B (2022). From evolution to revolution: accelerating crop domestication through genome editing. Plant Cell Physiol.

[CR54] Leebens-Mack JH, Barker MS, Carpenter EJ (2019). One thousand plant transcriptomes and the phylogenomics of green plants. Nature.

[CR55] Lei L, Goltsman E, Goodstein D (2021). Plant pan-genomics comes of age. Annu Rev Plant Biol.

[CR56] Lemmon ZH, Reem NT, Dalrymple J (2018). Rapid improvement of domestication traits in an orphan crop by genome editing. Nat Plants.

[CR57] Lewin HA, Robinson GE, Kress WJ (2018). Earth BioGenome Project: Sequencing life for the future of life. R Bot Gardens.

[CR58] Lewin HA, Richards S, Lieberman Aiden E (2022). The Earth BioGenome Project 2020: starting the clock. PNAS.

[CR59] Li YH, Zhou G, Ma J (2014). De novo assembly of soybean wild relatives for pan-genome analysis of diversity and agronomic traits. Nat Biotechnol.

[CR60] Li T, Yang X, Yu Y (2018). Domestication of wild tomato is accelerated by genome editing. Nat Biotechnol.

[CR61] Li W, Liu J, Zhang H (2022). Plant pan-genomics: recent advances, new challenges, and roads ahead. J Genet Genom.

[CR62] Liu HJ, Jian L, Xu J (2020). High-throughput CRISPR/Cas9 mutagenesis streamlines trait gene identification in maize. Plant Cell.

[CR63] Liu T, Zhang X, Li K (2023). Large-scale genome editing in plants: approaches, applications, and future perspectives. Curr Opin Biotechnol.

[CR64] Lu Y, Ye X, Guo R (2017). Genome-wide targeted mutagenesis in rice using the CRISPR/Cas9 system. Mol Plant.

[CR65] Lu X, Liu J, Ren W (2018). Gene-indexed mutations in maize. Mol Plant.

[CR66] Lughadha EN, Govaerts R, Belyaeva I (2016). Counting counts: Revised estimates of numbers of accepted species of flowering plants, seed plants, vascular plants and land plants with a review of other recent estimates. Phytotaxa.

[CR67] Ma X, Denyer T, Timmermans MCP (2020). PscB: A browser to explore plant single cell RNA-sequencing data sets. Plant Physiol.

[CR68] Marks RA, Hotaling S, Frandsen PB, VanBuren R (2021). Representation and participation across 20 years of plant genome sequencing. Nat Plants.

[CR69] Marsch-Martinez N, Greco R, Van Arkel G (2002). Activation tagging using the En-I maize transposon system in Arabidopsis. Plant Physiol.

[CR70] Matasci N, Hung LH, Yan Z (2014). Data access for the 1,000 Plants (1KP) project. Gigascience.

[CR71] Mccallum CM, Comai L, Greene EA, Henikoff S (2000). Targeted screening for induced mutations. Nat Biotechnol.

[CR72] McLay TGB, Murphy DJ, Holmes GD, Mathews S, Brown GK (2022). A genome resource for Acacia, Australia’s Largest Plant Genus. PLoS ONE.

[CR73] McWhite CD, Papoulas O, Drew K (2020). A pan-plant protein complex map reveals deep conservation and novel assemblies. Cell.

[CR74] Meng X, Yu H, Zhang Y (2017). Construction of a genome-wide mutant library in rice using CRISPR/Cas9. Mol Plant.

[CR75] Mergner J, Kuster B (2022). Annual review of plant biology plant proteome dynamics. Annu Rev Plant Biol.

[CR76] Mergner J, Frejno M, List M (2020). Mass-spectrometry-based draft of the Arabidopsis proteome. Nature.

[CR77] Meyer RS, Purugganan MD (2013). Evolution of crop species: genetics of domestication and diversification. Nat Rev Genet.

[CR78] Meyerowitz EM (2001). Prehistory and history of arabidopsis research. Plant Physiol.

[CR79] Michael TP (2014). Plant genome size variation: bloating and purging DNA. Brief Funct Genomics.

[CR80] Michael TP, VanBuren R (2020). Building near-complete plant genomes. Curr Opin Plant Biol.

[CR81] Michael TP, Jackson S (2013). The first 50 plant genomes. The Plant Genome.

[CR82] Mitsuda N, Takiguchi Y, Shikata M (2011). The new fioreDB database provides comprehensive information on plant transcription factors and phenotypes induced by CRES-T in ornamental and model plants. Plant Biotechnol.

[CR83] Muhammad T, Zhang F, Zhang Y, Liang Y (2019). RNA interference: a natural immune system of plants to counteract biotic stressors. Cells.

[CR150] Murphy KM, Ludwig E, Gutierrez J, Gehan MA (2024). Deep learning in image-based plant phenotyping. Ann Rev Plant Biol.

[CR84] Najera VA, Twyman RM, Christou P, Zhu C (2019). Applications of multiplex genome editing in higher plants. Curr Opin Biotechnol.

[CR85] Nesvizhskii AI (2014). Proteogenomics: concepts, applications and computational strategies. Nat Methods.

[CR86] Nolan TM, Shahan R (2023). Resolving plant development in space and time with single-cell genomics. Curr Opin Plant Biol.

[CR87] Ossowski S, Schwab R, Weigel D (2008). Gene silencing in plants using artificial microRNAs and other small RNAs. Plant J.

[CR88] Pan C, Li G, Bandyopadhyay A, Qi Y (2023). Guide RNA library-based CRISPR screens in plants: opportunities and challenges. Curr Opin Biotechnol.

[CR89] Pasha A, Shabari S, Cleary A, Chen X, Berardini T, Farmer A (2020). Araport lives: an updated framework for Arabidopsis bioinformatics. Plant Cell.

[CR90] Patel MK, Pandey S, Kumar M (2021). Plants metabolome study: emerging tools and techniques. Plants.

[CR91] Provart NJ, Alonso J, Assmann SM (2016). 50 years of Arabidopsis research: Highlights and future directions. New Phytol.

[CR92] Provart NJ, Brady SM, Parry G (2021). Anno genominis XX: 20 years of Arabidopsis genomics. Plant Cell.

[CR93] Rhee SY, Beavis W, Berardini TZ (2003). The Arabidopsis Information Resource (TAIR): A model organism database providing a centralized, curated gateway to Arabidopsis biology, research materials and community. Nucleic Acids Res.

[CR94] Rodríguez-Leal D, Lemmon ZH, Man J (2017). Engineering quantitative trait variation for crop improvement by genome editing. Cell.

[CR95] Rosso MG, Li Y, Strizhov N (2003). An *Arabidopsis thaliana* T-DNA mutagenized population (GABI-Kat) for flanking sequence tag-based reverse genetics. Plant Mol Biol.

[CR96] Samson F, Brunaud V, Balzergue S (2002). FLAGdb/FST: a database of mapped flanking insertion sites (FSTs) of Arabidopsis thaliana T-DNA transformants. Nucleic Acids Res.

[CR97] Saurabh S, Vidyarthi AS, Prasad D (2014). RNA interference: Concept to reality in crop improvement. Planta.

[CR98] Schauer N, Fernie AR (2006). Plant metabolomics: towards biological function and mechanism. Trends Plant Sci.

[CR99] Schwab R, Ossowski S, Riester M (2006). Highly specific gene silencing by artificial microRNAs in Arabidopsis. Plant Cell.

[CR100] Senior AW, Evans R, Jumper J (2020). Improved protein structure prediction using potentials from deep learning. Nature.

[CR101] Sessions A, Burke E, Presting G (2002). A high-throughput Arabidopsis reverse genetics system. Plant Cell.

[CR102] Seyfferth C, Renema J, Wendrich JR, Eekhout T, Seurinck R, Vandamme N (2021). Advances and opportunities in single-cell transcriptomics for plant research. Annu Rev Plant Biol.

[CR103] Sharma P, Al-Dossary O, Alsubaie B (2021). Improvements in the sequencing and assembly of plant genomes. GigaByte.

[CR104] Shen S, Zhan C, Yang C, Fernie AR, Luo J (2023). Metabolomics-centered mining of plant metabolic diversity and function: past decade and future perspectives. Mol Plant.

[CR105] Shendure J, Balasubramanian S, Church GM (2017). DNA sequencing at 40: Past, present and future. Nature.

[CR106] Soltis PS, Soltis DE (2021). Plant genomes: markers of evolutionary history and drivers of evolutionary change. Plants, People, Planet.

[CR107] Somssich M (2019). A short history of *Arabidopsis thaliana* (L.) Heynh. Columbia-0. PeerJ Prepr.

[CR108] Song YC, Das D, Zhang Y (2023). Proteogenomics-based functional genome research: approaches, applications, and perspectives in plants. Trends Biotechnol.

[CR109] Sreedasyam A, Plott C, Hossain MS (2023). JGI Plant Gene Atlas: an updateable transcriptome resource to improve functional gene descriptions across the plant kingdom. Nucleic Acids Res.

[CR110] Sun Y, Shang L, Zhu QH (2022). Twenty years of plant genome sequencing: achievements and challenges. Trends Plant Sci.

[CR111] Tadele Z (2016). Mutagenesis and TILLING to dissect gene function in plants. Curr Genomics.

[CR112] Tani H, Chen X, Nurmberg P (2004). Activation tagging in plants: a tool for gene discovery. Funct Integr Genomics.

[CR113] Tettelin H, Masignani V, Cieslewicz MJ (2005). Genome analysis of multiple pathogenic isolates of *Streptococcus agalactiae*: Implications for the microbial ‘pan-genome’. PNAS.

[CR114] Thibivilliers S, Libault M (2021). Plant Single-cell multiomics: cracking the molecular profiles of plant cells. Trends Plant Sci.

[CR115] Toufighi K, Brady SM, Austin R, Ly E, Provart NJ (2005). The botany array resource: e-Northerns, expression angling, and promoter analyses. Plant J.

[CR116] Tuskan GA, Difazio S, Jansson S (2006). The Genome of Black Cottonwood, *Populus trichocarpa* (Torr. & Gray). Science.

[CR117] van Dijk ADJ, Kootstra G, Kruijer W, de Ridder D (2021). Machine learning in plant science and plant breeding. iScience.

[CR118] Van Wijk KJ, Leppert T, Sun Q (2021). The Arabidopsis PeptideAtlas: harnessing worldwide proteomics data to create a comprehensive community proteomics resource. Plant Cell.

[CR119] van Wijk KJ, Leppert T, Sun Z (2024). Detection of the arabidopsis proteome and its post-translational modifications and the nature of the unobserved (Dark) proteome in PeptideAtlas. J Proteome Res.

[CR120] Vandenbussche M, Janssen A, Zethof J (2008). Generation of a 3D indexed Petunia insertion database for reverse genetics. Plant J.

[CR121] Vandenbussche M, Chambrier P, Bento SR, Morel P (2016). Petunia, your next supermodel?. Front Plant Sci.

[CR122] Velasco R, Zharkikh A, Troggio M (2007). A high quality draft consensus sequence of the genome of a heterozygous grapevine variety. PLoS ONE.

[CR123] Venter JC, Adams MD, Myers EW (2001). The sequence of the human genome. Science.

[CR124] Wada N, Ueta R, Osakabe Y, Osakabe K (2020). Precision genome editing in plants: state-of-the-art in CRISPR/Cas9-based genome engineering. BMC Plant Biol.

[CR125] Wang N, Long T, Yao W (2013). Mutant resources for the functional analysis of the rice genome. Mol Plant.

[CR126] Wang H, Cimen E, Singh N, Buckler E (2020). Deep learning for plant genomics and crop improvement. Curr Opin Plant Biol.

[CR127] Wang P, Wu X, Shi Z (2023). A large-scale proteogenomic atlas of pear. Mol Plant.

[CR128] Wei FJ, Droc G, Guiderdoni E, Hsing YC (2013). International consortium of rice mutagenesis: resources and beyond. Rice.

[CR129] Weigel D, Ahn JH, Blàzquez MA (2000). Activation Tagging in Arabidopsis. Plant Physiol.

[CR130] Wendrich JR, Yang BJ, Vandamme N, Verstaen K, Smet W, Van de Velde C (2020). Vascular transcription factors guide plant epidermal responses to limiting phosphate conditions. Science.

[CR131] Willems P, Ndah E, Jonckheere V (2017). N-terminal proteomics assisted profiling of the unexplored translation initiation landscape in *Arabidopsis thaliana*. Mol Cell Proteom.

[CR132] Willems P, Ndah E, Jonckheere V (2022). To new beginnings: riboproteogenomics discovery of N-terminal proteoforms in *Arabidopsis thaliana*. Front Plant Sci.

[CR133] Winter D, Vinegar B, Nahal H (2007). An “electronic fluorescent pictograph” Browser for exploring and analyzing large-scale biological data sets. PLoS ONE.

[CR134] Woody ST, Austin-Phillips S, Amasino RM, Krysan PJ (2007). The WiscDsLox T-DNA collection: An arabidopsis community resource generated by using an improved high-throughput T-DNA sequencing pipeline. J Plant Res.

[CR135] Xanthopoulou A, Moysiadis T, Bazakos C (2022). The perennial fruit tree proteogenomics atlas: a spatial map of the sweet cherry proteome and transcriptome. Plant J.

[CR200] Xie L, Gong X, Yang K (2024). Technology-enabled great leap in deciphering plant genomes. Nat Plants.

[CR136] Xu Y, Liu X, Cao X (2021). Artificial intelligence: a powerful paradigm for scientific research. The Innovation.

[CR137] Yu H, Li J (2022). Breeding future crops to feed the world through de novo domestication. Nat Commun.

[CR138] Yu J, Hu S, Wang J (2002). A Draft Sequence of the Rice Genome (*Oryza sativa* L. ssp. indica). Science.

[CR139] Yu X, Yang D, Guo C, Gao L (2018). Plant phylogenomics based on genome-partitioning strategies: progress and prospects. Plant Divers.

[CR140] Yu H, Lin T, Meng X (2021). A route to de novo domestication of wild allotetraploid rice. Cell.

[CR141] Yu Y, Zhang H, Long Y (2022). Plant public RNA-seq database: a comprehensive online database for expression analysis of ~45 000 plant public RNA-Seq libraries. Plant Biotechnol J.

[CR142] Yu X, Liu Z, Sun X (2023). Single-cell and spatial multi-omics in the plant sciences: technical advances, applications, and perspectives. Plant Commun.

[CR143] Zaidi SSEA, Mahas A, Vanderschuren H, Mahfouz MM (2020). Engineering crops of the future: CRISPR approaches to develop climate-resilient and disease-resistant plants. Genome Biol.

[CR144] Zheng D, Xu J, Lu Y (2023). Recent progresses in plant single-cell transcriptomics. Crop Design.

[CR145] Zhou J, Li D, Wang G (2020). Application and future perspective of CRISPR/Cas9 genome editing in fruit crops. J Integr Plant Biol.

[CR146] Zhu FY, Chen MX, Ye NH (2017). Proteogenomic analysis reveals alternative splicing and translation as part of the abscisic acid response in Arabidopsis seedlings. Plant J.

[CR147] Zhu H, Li C, Gao C (2020). Applications of CRISPR–Cas in agriculture and plant biotechnology. Nat Rev Mol Cell Biol.

[CR148] Zimmermann P, Hirsch-Hoffmann M, Hennig L, Gruissem W (2004). GENEVESTIGATOR. Arabidopsis microarray database and analysis toolbox. Plant Physiol.

[CR149] Zsögön A, Čermák T, Naves ER (2018). De novo domestication of wild tomato using genome editing. Nat Biotechnol.

